# Circulating CD117+/CD34+/CD45dim Myeloblasts as Biomarkers of Disease Activity in Primary Myelofibrosis—A Pilot Study

**DOI:** 10.1002/jha2.70304

**Published:** 2026-05-14

**Authors:** Malak Tamer Abdelmaksoud, Zsuzsanna Hevessy, Judit Bedekovics, Gábor Méhes, Árpád Illés, Zsófia Simon, László Imre Pinczés

**Affiliations:** ^1^ Division of Hematology Department of Internal Medicine Faculty of Medicine University of Debrecen Debrecen Hungary; ^2^ Department of Laboratory Medicine Faculty of Medicine University of Debrecen Debrecen Hungary; ^3^ Department of Pathology Faculty of Medicine University of Debrecen Debrecen Hungary; ^4^ Doctoral School of Clinical Medicine University of Debrecen Debrecen Hungary

**Keywords:** circulating myeloblast, flow cytometry, myelofibrosis, prognosis

## Abstract

**Introduction:**

Clinical benefit measures and surrogates for leukaemic transformation or overall survival are scarce in MF. Circulating myeloblast (CMB) counts have the biological plausibility to indicate disease evolution, but they are still determined by an outdated approach with microscopic examination and fixed cut‐offs. Our aim was to evaluate the prognostic value of CD117+/CD34+/CD45dim CMBs (CMB‐FC) as continuous variables in MF patients at diagnosis and during cytoreductive therapy.

**Methods:**

Absolute counts (CMB‐FC#) and relative percentages (CMB‐FC%) were determined using multiparameter flow cytometry (FC). Associations of CMB‐FCs and well‐established prognostic markers of MF were determined by linear regression and non‐parametric tests. Longitudinal CMB‐FC changes were analysed using linear mixed‐effects models.

**Results:**

In the 29 analysed patients, meaningful correlations were observed between CMB‐FCs and blood counts, *JAK2* VAF and LDH levels among several other markers. Patients with overt MF had higher CMB‐FC# and CMB‐FC% compared to those with a pre‐fibrotic histological subtype, while CMB‐FC# increased gradually with fibrosis grade and within well‐established prognostic scores. No significant effect on CMB‐FC levels was observed over time in response to treatment.

**Conclusion:**

This novel approach for quantifying CMBs has strong potential to reflect disease activity and prognosis in MF.

Trial Registration: The authors have confirmed clinical trial registration is not needed for this submission

## 1 Introduction

Myelofibrosis (MF) is a haematopoietic stem‐cell‐derived myeloproliferative neoplasm (MPN) that adversely affects quality of life, increases the risk of progression to acute myeloid leukaemia (AML) and shortens overall survival [1]. MF is classified into primary (PMF) and secondary (SMF) forms, the latter arising from antecedent polycythaemia vera (PV) or essential thrombocythaemia (ET). PMF is further subcategorized into pre‐fibrotic and overt forms, based on the extent of bone marrow fibrosis [2].

The major pathophysiological hallmark of MF is the hyperactivated Janus Kinase–Signal Transducers and Activators of Transcription (*JAK‐STAT*) pathway, driven by altered *JAK2*, *CALR* or *MPL* signalling, leading to progressive bone marrow fibrosis, extramedullary haematopoiesis and a heterogeneous disease burden. Leukaemic transformation remains the most adverse disease outcome, occurring in approximately 10%–20% of patients within 10 years of diagnosis and representing the major cause of MF‐related mortality [3]. Disease progression is a major contributor to MF‐related mortality, highlighting the importance of risk stratification and personalized therapeutic approaches.

Current drug therapies, including *JAK* inhibitors and hydroxycarbamide, remain palliative in essence, as their impact on overall survival remains limited and subject to ongoing debate, while they do not significantly alter the disease's natural progression [2]. Allogeneic haematopoietic stem cell transplantation remains the sole curative approach, yet its use is limited by substantial treatment‐related morbidity and mortality [4]. Considering these limitations, precise patient stratification is essential to facilitate the treatment decision‐making process.

Traditional prognostic models in MF assessed clinical parameters and laboratory variables. Advances in understanding of disease biology have led to the incorporation of genetic and molecular data, resulting in the development of integrated prognostic scoring systems, including Dynamic International Prognostic Scoring System (DIPSS), Mutation‐Enhanced IPSS (MIPSS70) and Genetically Inspired PSS (GIPSS) for PMF and Myelofibrosis Secondary to PV and ET‐Prognostic Model (MYSEC‐PM) for SMF [5, 6, 7, 8].

Integration of meaningful clinical parameters into prognostic scoring systems is the cornerstone of precise risk stratification and, therefore, adequate therapeutic decision‐making. Circulating myeloblasts (CMBs) reflect the dynamic interplay between bone marrow fibrosis and extramedullary haematopoiesis and therefore represent a biologically plausible surrogate marker of disease activity. Nevertheless, in the era of modern molecular testing methods, CMB percentage continues to be determined by morphological evaluation. The merits of this method are undermined, in part, by the limitations of visual differentiation between immature myeloid precursors and the resulting interobserver variability, and also by the significantly lower number of detected events compared to molecular methods. As the absolute number of circulating CD34+ cells determined by multiparameter flow cytometry (FC) has been proven to correlate with the CMB count, several promising attempts have been made to determine its individual prognostic value in MF, regarding discrimination from other MPNs and survival [9, 10, 11, 12, 13, 14, 15, 16]. However, no attempts have yet been made to precisely determine the number of CMBs using an appropriate FC panel and assess its potential value as a biomarker in PMF.

In this study, we aimed to address these methodological limitations by employing FC to quantify circulating CD117+/CD34+/CD45dim cells (CMB‐FC), corresponding to peripheral blood myeloblasts, at diagnosis and during the disease course in PMF. We evaluated the association of CMB‐FC with established prognostic markers at baseline and during drug therapy to assess its biological and clinical relevance as a marker of disease activity.

## 2 Methods

### 2.1 Patients

We retrospectively analysed the demographic and clinical data of adult patients with PMF treated at the University of Debrecen between January 2022 and December 2024. All patients had a histologically confirmed diagnosis of PMF in line with the 2022 WHO criteria and underwent FC analysis at diagnosis and during at least one follow‐up assessment. FC was routinely performed at baseline, at 3 and 6 months after the initiation of first‐line therapy, and every 6 months thereafter.

Complete blood count and serum chemistry data were available at diagnosis or within one month prior to treatment initiation. Laboratory anaemia was determined as a haemoglobin concentration below the lower limit of normal value of < 115 g/L in women and < 130 g/L in men. Transfusion dependency was defined as symptomatic anaemia requiring red blood cell transfusion at diagnosis.

Thrombocytopenia was defined as platelet count < 100 × 10^9^/L, while leukocytosis was defined as white blood count > 11 × 10^9^/L. Spleen size was measured at each visit as the distance below the left costal margin. Risk stratification was performed at the time of diagnosis according to the DIPSS, MIPSS70 and GIPSS formulas. Patients were followed until death or the last follow‐up. As no progression events occurred during the study period, two separate case studies of disease progression were evaluated to explore CMB‐FC dynamics preceding clinical transformation. The study was approved by the institutional review board of the University of Debrecen (IRB number: 7154) and conducted in accordance with the Declaration of Helsinki. Written informed consent was obtained from all participants.

### 2.2 Flow Cytometry and Molecular Analysis

Multiparametric eight‐colour FC was performed using pre‐titrated mouse anti‐human monoclonal antibodies. A total of 300,000 events were recorded, corresponding to a limit of detection of 0.0033% and a limit of quantitation of 0.01%. Circulating myeloblast percentage (CMB‐FC%) was calculated as CD117+/CD34+/CD45dim cells divided by total CD45+ cells. Absolute circulating myeloblast count (CMB‐FC#) was calculated using a dual‐platform approach: CMB‐FC# = (WBC × 1000 × CMB − FC%) / 100.

Mutation analysis was performed on peripheral blood DNA, including *JAK2 V617F*, *CALR Exon 9* and *MPL W515* mutations, which were detected by real‐time polymerase chain reaction or high‐resolution melting analysis, while high‐risk mutations were determined by next‐generation sequencing. Detailed FC and molecular methods are provided in the Supporting Information.

### 2.3 Treatment

Patients were treated according to the evidence and consensus‐based practice guidelines of the Hungarian Society of Hematology and Transfusion, consistent with current international guidelines [17]. Ruxolitinib treatment was indicated for the treatment of disease‐related splenomegaly or symptoms in patients with intermediate‐ or high‐risk disease profiles. Hydroxycarbamide was the first‐line drug of choice for MF‐associated leukocytosis or thrombocytosis in the absence of marked splenomegaly or constitutional symptoms.

### 2.4 Statistical Analysis

Categorical variables were summarised as frequencies and percentages, and continuous variables as medians and ranges. Data normality was assessed using the Kolmogorov–Smirnov test.

Group comparisons were performed using *t*‐test or Mann–Whitney *U* test, as appropriate, and Kruskal–Wallis test for comparison of three or more groups. Associations between CMB‐FC and disease markers were evaluated using simple linear regression.

Longitudinal changes in CMB‐FC# and CMB‐FC% were analysed using linear mixed‐effects models (LMM) to account for repeated measures and missing data. Time was treated as a continuous variable (0, 3, 6 and 12 months), and patients were included as random effects, allowing for individual variations in biomarker trajectories. To explore potential nonlinear effects, we extended the model by adding a quadratic term. To ensure the validity of the models, Little's MCAR test was conducted to examine whether missing data were missing completely at random (MCAR). Statistical analyses were performed using Stata 18.0 (StataCorp LLC, Texas, USA), with *p* < 0.05 considered significant.

## 3 Results

### 3.1 Baseline Assessment

**FIGURE 1:**
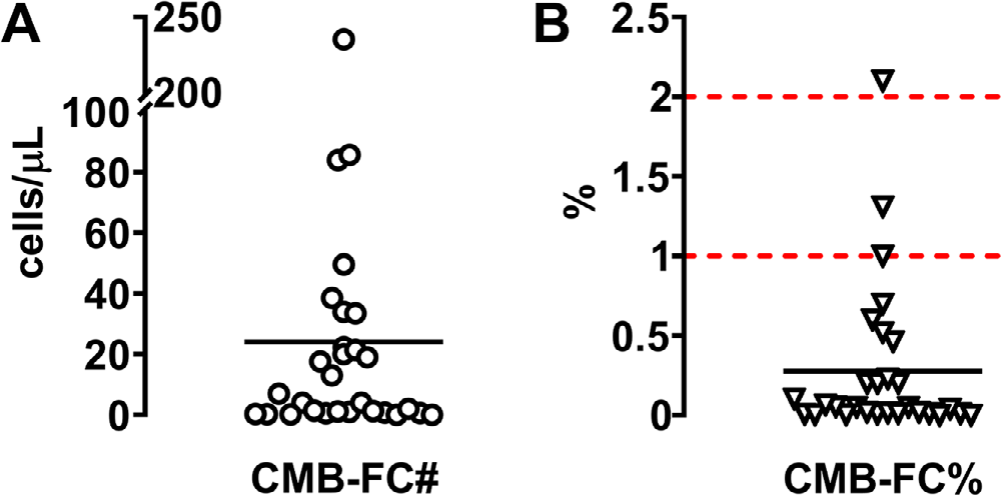
Mean values of absolute and relative counts of circulating myeloblasts determined by flow cytometry. The straight lines represent the mean, while the dotted lines indicate the traditional cut‐off levels. CMB‐FC#, absolute circulating myeloblast count; CMB‐FC%, relative circulating myeloblast count.

Twenty‐nine patients had sufficient laboratory and clinical data for analysis. Baseline characteristics are summarized in Table 1. At diagnosis, median and mean CMB‐FC# were 4 and 24.08 cells/µL, respectively, while median and mean CMB‐FC% were 0.05% and 0.28%. The distribution was right‐skewed, with most patients exhibiting low levels of CMBs, and a subset presenting with markedly elevated values. Notably, mean CMB‐FC% remained well below traditional morphological cut‐offs (1%–2%) (Figure 1).

**TABLE 1:** Patient characteristics.

Characteristics	Data
Age, median, years (range)	70 (32–85)
Myelofibrosis subtype, *n* (%)	
Pre‐fibrotic	13 (45%)
Overt	16 (55%)
Fibrosis grade, *n* (%)	
0	4 (14%)
1	9 (31%)
2	7 (24%)
3	7 (24%)
Unknown	2 (7%)
Driver mutation, *n* (%)	
*JAK2*	23 (79%)
*CALR*	1 (3%)
*MPL*	3 (11%)
Triple negative	2 (7%)
Splenomegaly, *n* (%)	17 (59%)
Anaemia, *n* (%)	9 (31%)
Transfusion dependency, *n* (%)	6 (21%)
Thrombocytopenia, *n* (%)	5 (17%)
Leucocytosis, *n* (%)	15 (52%)
LDH, median, U/L (range)	286 (148–1424)
Ferritin, median, µg/L (range)	165 (10–1869)
MIPSS risk status, *n* (%)	
Low	9 (31%)
Intermediate	15 (52%)
High	5 (17%)
GIPSS risk status, *n* (%)	
Low	9 (31%)
Intermediate‐1	13 (45%)
Intermediate‐2	12 (41%)
High	2 (7%)
Unknown	1 (3%)

Abbreviations: *CALR*, calreticulin; GIPSS, Genetically Inspired Prognostic Scoring System; *JAK2*, Janus kinase 2; LDH, lactate dehydrogenase; MIPSS70, Mutation‐Enhanced International Prognostic Score System; *MPL*, myeloproliferative leukaemia virus oncogene

CMB‐FC% was significantly higher in patients with laboratory anaemia compared to non‐anaemic patients (*p* = 0.045) and was significantly elevated in transfusion‐dependent patients (*p *< 0.001). Negative correlations were observed between CMB‐FC% and haemoglobin (*R* ^2^ = 0.215, *p* = 0.011), as well as thrombocyte count (*R* ^2^ = 0.181, *p* = 0.022), representing a link between increased myeloblast burden and cytopenias.

Disease biology markers have also been associated with CMB‐FCs. Greater clonal burden measured with *JAK2* VAF positively correlated with CMB‐FC# (*R* ^2^ = 0.172, *p* = 0.049) in *JAK2*‐mutated patients. Patients with overt MF had higher CMB‐FC# and CMB‐FC% compared to those with a pre‐fibrotic histological subtype (*p* = 0.005 and *p* = 0.015, respectively). CMB‐FC# increased significantly with fibrosis grade (Kruskal–Wallis *p* = 0.001), indicating a direct relationship with worsening bone marrow fibrosis. LDH levels positively correlated with both CMB‐FC# (R^2^ = 0.378, *p* < 0.001) and CMB‐FC% (R^2^ = 0.561, *p* < 0.0001), while ferritin levels positively correlated with CMB‐FC% (R^2^ = 0.208, *p* = 0.043). The myeloid‐to‐erythroid (M:E) ratio of the bone marrow positively correlated with CMB‐FC# (R^2^ = 0.151, *p* = 0.045).

**FIGURE 2:**
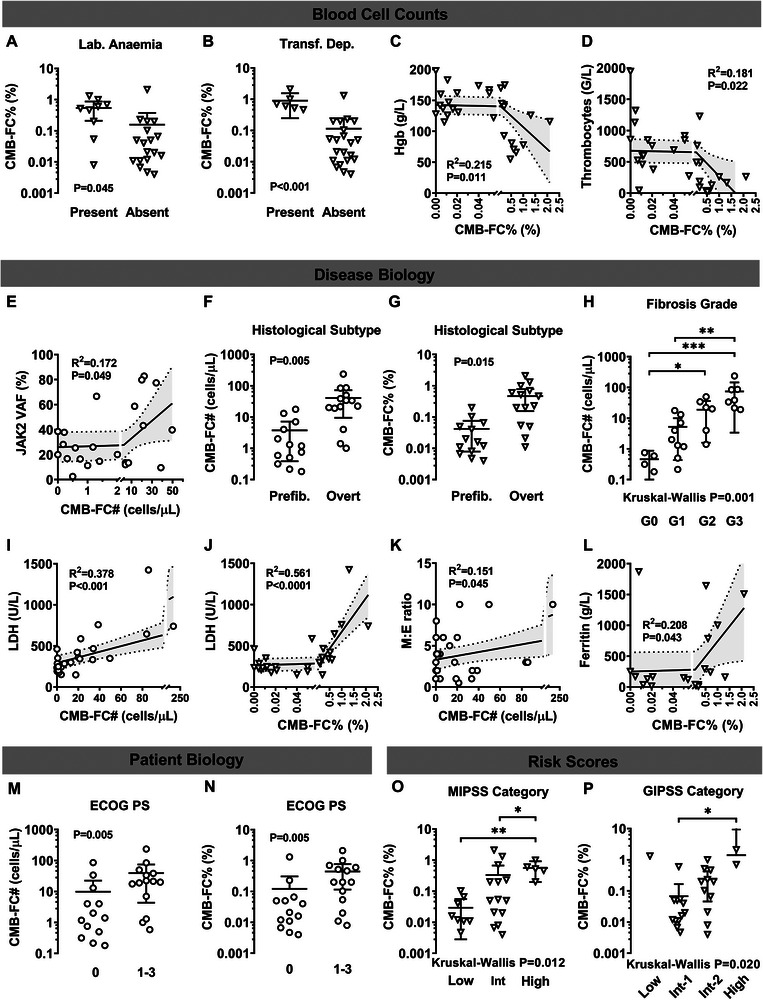
Associations of markers of disease severity and circulating myeloblast counts determined by flow cytometry. On the scatter plots (A, B, F–H and M–P) the lines represent the mean value and the 95% confidence intervals. At simple linear regression analysis (C–E and I–L) the regression line (straight lines) with 95% confidence intervals (dotted lines) are indicated. CMB‐FC#, absolute circulating myeloblast count; CMB‐FC%, relative circulating myeloblast count; Hgb, haemoglobin; *JAK2*, Janus kinase 2; VAF, variant allele frequency; Prefib., pre‐fibrotic; G, grade; LDH, lactate dehydrogenase; M:E, myeloid‐to‐erythroid; ECOG PS, Eastern Cooperative Oncology Group Performance Status; MIPSS70, Mutation‐Enhanced International Prognostic Score System; GIPSS, Genetically Inspired Prognostic Scoring System.

Higher Eastern Cooperative Oncology Group (ECOG) Performance Status was associated with increased CMB‐FC# and CMB‐FC% (*p* = 0.005 for both). CMB‐FC% increased significantly across MIPSS70 and GIPSS risk categories (Kruskal–Wallis *p* = 0.012 and *p* = 0.020, respectively). Associations between CMB‐FC counts and markers of disease severity are depicted in Figure 2.

### 3.2 Longitudinal Assessment

**FIGURE 3:**
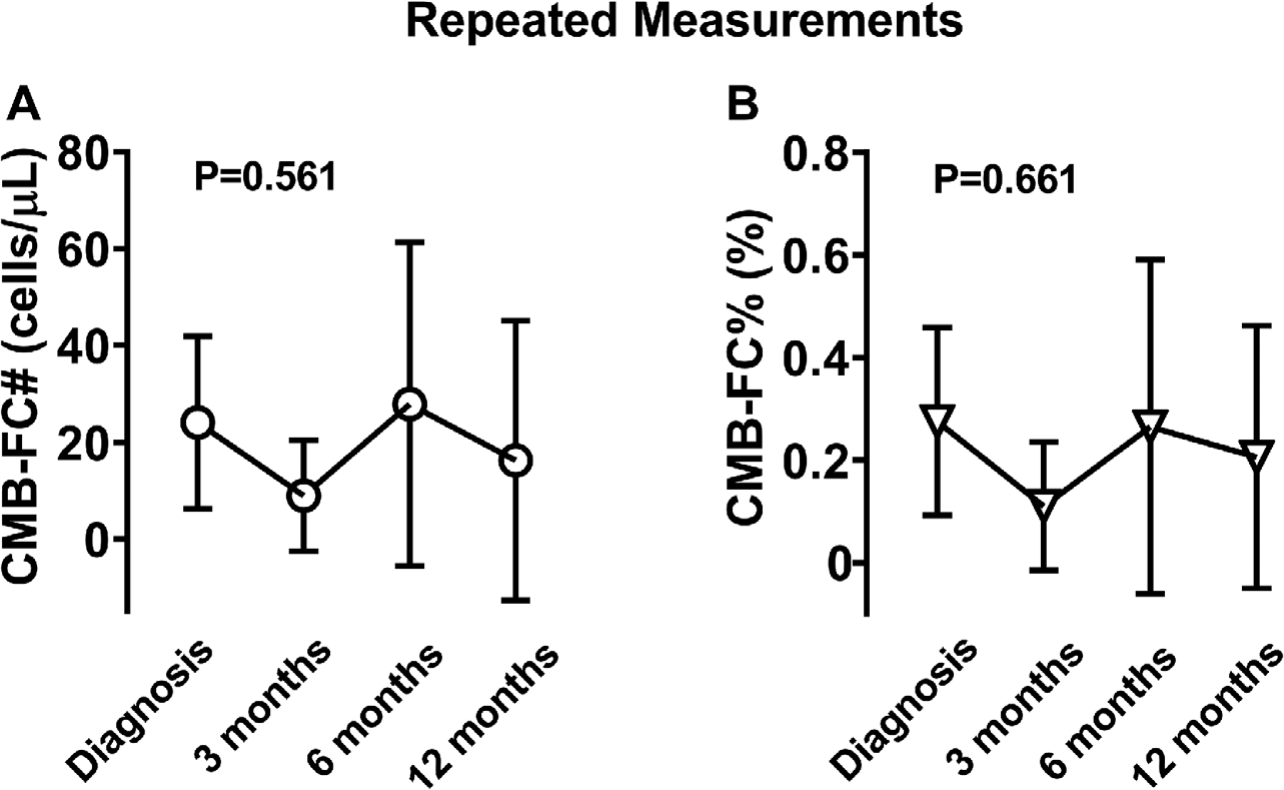
Longitudinal assessment of circulating myeloblast counts. Mean values (circle) are connected with straight lines. Error bars represent the 95% Confidence Intervals. CMB‐FC#, absolute circulating myeloblast count; CMB‐FC%, relative circulating myeloblast count.

A linear mixed‐effects model indicated no significant effect on CMB‐FC# levels over time in response to treatment (*β*1 = 0.60, *p* = 0.561) (Figure 3A). Inclusion of a quadratic term did not improve model fit, indicating that CMB‐FC# followed a stable trajectory. Significant random intercept variance suggested considerable between‐subject variability in baseline CMB‐FC# levels. Similarly, CMB‐FC% showed no significant temporal trend (*β*1 = 0.002, *p* = 0.661), despite baseline variability (*β*0 = 0.2856, *p* = 0.002) (Figure 3B). Little's MCAR test confirmed that missing data were MCAR (*p *> 0.05), indicating that missing values were randomly distributed and did not introduce systematic bias.

**FIGURE 4:**
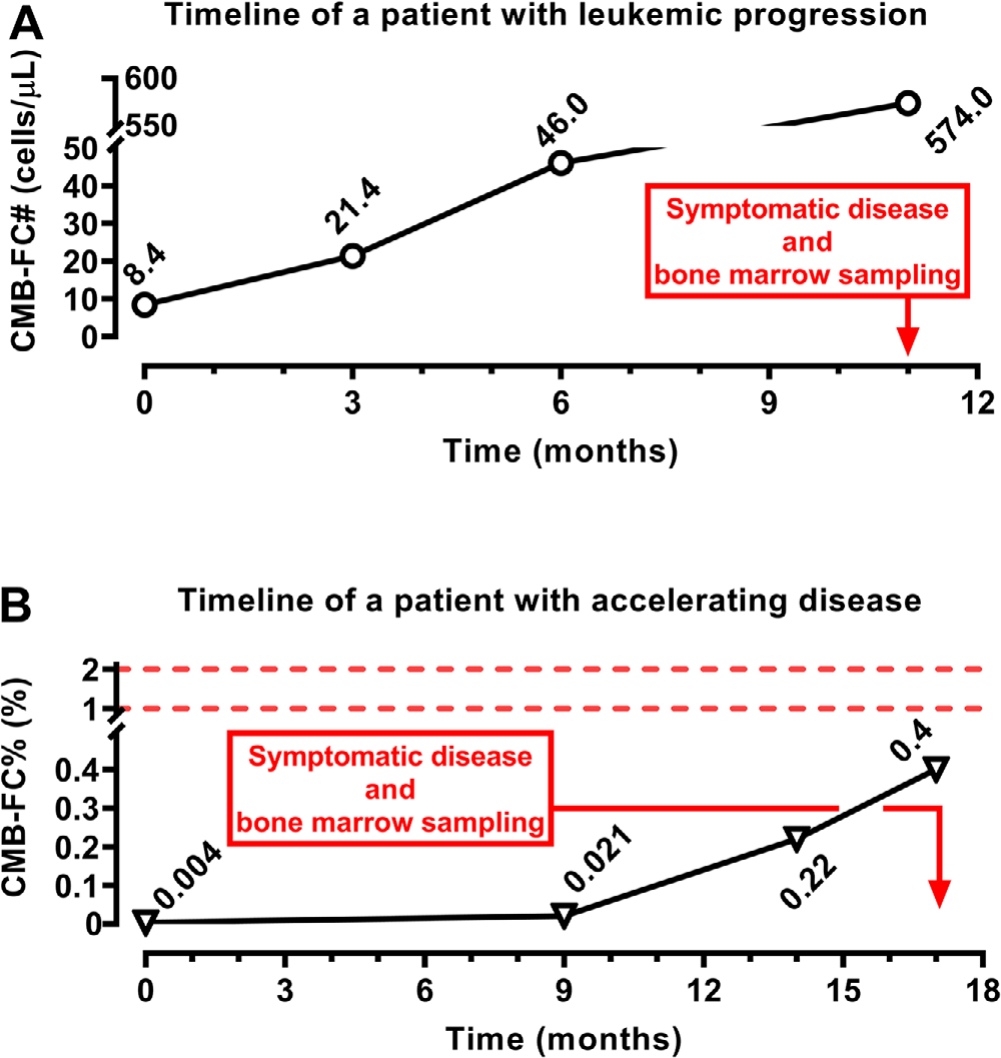
Change in circulating myeloblast counts during disease progression (A, B). The dotted lines indicate the traditional cut‐off levels. CMB‐FC#, absolute circulating myeloblast count; CMB‐FC%, relative circulating myeloblast count.

As no progression events occurred within the main cohort during follow‐up, we evaluated two distinct cases of disease progression in patients who were not fit for evaluation in the study population due to missing baseline data. In the first case, a patient who progressed to leukaemic transformation exhibited a steady increase in CMB‐FC# from 8.4 to 574 cells/µL over 11 months, with symptomatic progression only occurring at the final time point (Figure 4A). In the second case, a patient with an accelerating disease profile (increase in MIPSS70 risk category and ruxolitinib failure) showed an increase in CMB‐FC% from 0.004% to 0.4% over 17 months, preceding clinical deterioration (Figure 4B). Notably, these remarkable increases occurred below the conventional 1% cut‐off.

## 4 Discussion

Over the past decade, clinical benefit measures for *JAK* inhibitor therapy have become integral to MF clinical trials. While current drug therapy has a specific impact on reducing symptom burden and splenomegaly, its effect on the natural history of the disease remains limited [1, 2]. Therefore, while ≥ 50% reduction in total symptom score (TSS50) and ≥ 35% reduction in spleen volume (SVR35) are excellent at capturing clear improvements in quality of life, they are specifically designed to measure the symptomatic relief provided by *JAK* inhibitors rather than track long‐term disease evolution. Therefore, to develop treatments that significantly improve survival, a reassessment of key efficacy endpoints is necessary [18]. In the current landscape of MF treatment, an ideal biomarker would reflect the severity of the disease at the time of diagnosis, with levels indicating disease progression before the onset of clinical symptoms. Potential candidates for this approach are the VAF of driver mutations and the number of CMBs, as they have the biological plausibility to directly reflect disease burden.

Changes in VAFs correlate with response and outcomes in other MPNs, and their clinical utility has been demonstrated in MF patients undergoing allogeneic haematopoietic stem cell transplantation [19, 20]. Although the allele burden reductions in most patients treated with ruxolitinib were gradual over the course of previous reports and were similar across MF subtypes, these changes were not significant [21, 22]. Also, *JAK* inhibitor monotherapy trials have not reported an association between VAF burden and leukaemia‐free or overall survival as primary endpoints.

CMBs play a significant role in the commonly used prognostic models for patients with MF. However, these conventional risk models use arbitrary thresholds (≥1%–2%) to stratify risk, potentially oversimplifying prognostic implications. The adoption of these cut‐offs is based on a number of studies that share both a methodology based on microscopic evaluation and the identification of outliers as risk factors (Table S1) [6, 7, 8, 9, 10, 11, 12, 13, 14, 15, 16, 23, 24, 25, 26, 27, 28, 29, 30, 31, 32, 33, 34, 35, 36, 37, 38, 39, 40, 41, 42, 43, 44]. This approach may have inherent limitations, as subjective microscopic assessments are susceptible to interobserver variability and limited detection sensitivity, particularly in cases with low myeloblast percentages. While these results have been important in highlighting the poor prognostic role of markedly elevated CMB%, they do not help in stratifying patients who have dynamic changes in their CMB values well below the 1% range. Based on trends in recent decades, visual analysis reached the lower limits of detection and has proven to be a significant predictor in numerous studies at this level, which observation also indicates that it is worth pushing the detection limit downward using molecular methods (Figure S1).

An FC‐enhanced model by Mannelli et al. has already been proven to increase the performance of conventional risk stratification regarding IPSS and MIPSS70+ in PMF [14]. Integrating their scoring system into existing prognostic models significantly improved their ability to predict overall survival, with a notable fraction of patients (up to 30% for IPSS and 13% for MIPSS70+) being reassigned to different risk categories, emphasizing the importance of molecular characterization of CMB counts. Iurlo et al. employed FC to quantify circulating CD34+ levels in patients with PMF or SMF over the course of ruxolitinib treatment, experiencing a steady decline in CD34+ cell counts that closely paralleled the reduction in spleen size [15]. Previous studies of by Barosi et al., Arora et al. and Demoy et al. identified different CD34+ cut‐offs for increased risk regarding overall‐ and leukaemia‐free survival (50–300 × 10^6^ cells/L), while Alchalby et al. argued about the confirmatory value of elevating CD34+ counts for progression after allo‐HCT [9, 11, 13, 16]. Collectively, these studies have highlighted the role of FC and FC‐enhanced models in refining prognostic stratification in MF. However, they leave room for improvement in further research, as the number of CD34+ cells is related to peripheral blasts, but cannot be considered equivalent to them.

Our approach with the precise identification of CMB‐FCs as CD117+/CD34+/CD45dim circulating cells proved that CMB‐FC# and CMB‐FC% at diagnosis correlate with known markers of disease activity in MF. As the severity of anaemia and thrombocytopenia in MF is associated with shorter overall‐ and leukaemia‐free survival, and reduced quality of life, our findings add new insight by revealing a significant correlation between decreasing cytopenias and increased CMB‐FC burden [45, 46]. Beyond haematological parameters, our findings also highlight the relationship between disease biology and CMB‐FC counts. Several studies showed that the *JAK2* mutation burden may have significant clinical implications, such as a greater likelihood of disease progression and reduced overall survival [47]. Expanding on these findings, we observed that patients with a higher mutant *JAK2* VAF tended to have elevated CMB‐FC#, suggesting a potential link between clonal burden and myeloblast expansion. The elevation of CMB‐FC# and CMB‐FC% in patients with overt PMF compared to pre‐fibrotic histological subtype and the steady increase in CMB‐FC# through fibrosis grades support the hypothesis that progressive bone marrow fibrosis is associated with translocation of myeloblasts into the circulation. This link is further strengthened by a positive correlation between the M:E ratio of the bone marrow and CMB‐FC#. Disease symptomatology also has the potential to be reflected by CMB counts, reinforced by the relationship of CMB‐FC# and CMB‐FC% with disease‐specific cytokine activity markers and ECOG status. Finally, the elevated CMB‐FC% levels of patients with high MIPSS70 and GIPSS risk scores suggest that incorporating CMB‐FC assessment as a continuous variable into prognostic models may enhance their precision.

While current treatments do not modify disease biology, we expected CMB‐FCs to remain stable or increase over time. Accordingly, in our study, neither biomarker exhibited a statistically significant temporal change, as patients who were given drug therapy showed no modification in their CMB‐FC counts during 12 months of treatment. As no significant fixed effects of time were detected for either biomarker, our findings reinforce the prevailing understanding that existing treatments do not modify disease biology.

The relatively short median follow‐up (1.49 years) limits the ability to assess leukaemic transformation and long‐term disease evolution; therefore, the longitudinal findings should be interpreted in the context of early disease dynamics rather than progression endpoints. As there were no progressive events in the study population, we reviewed the CMB‐FC dynamics of two separate patients. In these cases, CMB‐FC# and CMB‐FC% trajectories reflected the natural disease course in an evolving manner. CMB‐FC# rise preceded clinical leukaemic transformation, suggesting it could serve as an early indicator of impending blast‐phase MF. Also, CMB‐FC% increased before clinical progression, with early shifts occurring far below the 1% cut‐off.

The limit of detection is probably the most important aspect of FC analysis. Our study demonstrates that the majority of patients have a CMB‐FC% an order of magnitude lower than the detection limit of visual inspection. This suggests that even minimal myeloblast presence may be meaningful in the context of disease progression and that the traditional cut‐offs are not representative of the patient population. Our findings clearly indicate that there is potential for large, clinically relevant changes in CMB‐FCs, reinforcing the idea that these early warning biomarkers may have predictive utility beyond traditional thresholds.

Our study is subject to inherent limitations common to retrospective studies, such as missing or inconsistent records. To mitigate this, we only included patients with sufficient laboratory and clinical data at diagnosis, and missing data during follow‐up have been properly accounted for using the appropriate statistical methods. It should be noted that the use of a predefined immunophenotypic definition of myeloblasts may not capture highly aberrant blast populations, particularly in the context of leukaemic transformation; however, the use of a standardized gating strategy was essential to ensure reproducibility and longitudinal comparability. Although many positive correlations were discovered between markers of disease severity and both CMB‐FC# and CMB‐FC%, some correlations arose with only one of these markers. This discordance may stem from the influence of different treatments leading to heterogeneity in the patient's blood cell counts, thereby distorting the determination of percentages. Whether the absolute or relative count of CMB‐FCs is more representative of the disease biology of a patient has not been further evaluated in this study. However, we believe that our findings provide substantial ground for a more precise analysis of the prognostic value of CMB‐FC# and CMB‐FC% and their relationship with other disease markers and meaningful outcomes.

## 5 Conclusion

We conclude that the majority of MF patients do not benefit from the current approach to CMB measurement. A novel method for the evaluation of CMB‐FC# and CMB‐FC% provides valid biomarkers of disease activity at diagnosis of MF and also has the potential to recognize natural disease evolution. We recommend retrospectively investigating CMB‐FC# and CMB‐FC% in other centres to further evaluate their prognostic potential, incorporate them into currently used prognostic scores, and prospectively investigate them as surrogate markers for efficacy and disease progression in clinical trials for the treatment of MF.

## Author Contributions

M.T.A. and L.I.P. were responsible for data collection, statistical analysis and creating the manuscript. M.T.A., Á.I., Z.S. and L.I.P. were responsible for study design. Z.H. was responsible for multicolour flow cytometry analysis. J.B. and G.M. were responsible for histological analysis. All authors read and approved the final manuscript.

## Funding

The authors have nothing to report.

## Conflicts of Interest

The author declares no conflicts of interest.

## Supporting information




**Supporting File 1**: jha270304‐sup‐0001‐SuppMat.docx

## Data Availability

The datasets used and analysed during the current study are available from the corresponding author on reasonable request.
