# Supplementary material for: Circulating CD117+/CD34+/CD45dim Myeloblasts as Biomarkers of Disease Activity in Primary Myelofibrosis—A Pilot Study
Source: EJHaem. 2026 May 14;7(3):e70304. doi: 10.1002/jha2.70304 (PMC13172770; doi:10.1002/jha2.70304)
Supplement: Supplementary file 1 — Supporting File 1: jha270304‐sup‐0001‐SuppMat.docx [file JHA2-7-e70304-s001.docx]

**Supplementary material**

**of**

**Circulating CD117+/CD34+/CD45dim myeloblasts as biomarkers of disease activity in primary myelofibrosis – a pilot study**

Malak Tamer ABDELMAKSOUD^1^, Zsuzsanna HEVESSY^3^, Judit BEDEKOVICS^4^, Gábor MÉHES^4^, Árpád ILLÉS^1,2^, Zsófia SIMON^1,2^, László Imre PINCZÉS^1,2^

^1^ Division of Hematology, Department of Internal Medicine, Faculty of Medicine, University of Debrecen, Debrecen, Hungary, ^2^ Doctoral School of Clinical Medicine, University of Debrecen, Debrecen, Hungary, ^3^ Department of Laboratory Medicine, Faculty of Medicine, University of Debrecen, Debrecen, Hungary, ^4^ Department of Pathology, Faculty of Medicine, University of Debrecen, Debrecen, Hungary

Supplementary Figures: 1

Supplementary Tables: 1

Supplementary Methods

*Flow cytometry and molecular analysis*

Multiparametric eight-colour flow cytometric assessment was performed using a panel of pre-titrated mouse anti-human monoclonal antibodies (mAb), including CD7, CD33, CD34, CD38, CD45, CD56, CD71, CD117 and HLA DR. These monoclonal antibodies were combined in one tube to characterize myeloblasts. Stain, lyse and wash protocol was used to perform cell surface staining. Briefly, 50 uL of ethylenediaminetetraacetic acid tripotassium salt (K3-EDTA) anticoagulated peripheral whole blood was mixed with the cocktail of mAbs in a FC tube and incubated for 15 minutes in dark at room temperature (20-22°C). After incubation, 1 mL of 10x BD FACS Lysing Solution lysis buffer (Becton, Dickinson and Company - BD, Franklin Lakes, NJ, USA) was added and incubated for 10 more minutes. Upon incubation tubes were washed once with 1 mL of phosphate-buffered saline and centrifugation at 300 g for 5 minutes, the pellet was finally resuspended in 400 uL of 1% paraformaldehyde. All samples were stained within eight hours of sample collection and upon flow cytometric staining, 1% paraformaldehyde was added to fix the cells for acquisition and analysis. Sample acquisition was performed using a FACSCanto II flow cytometer (BD, Franklin Lakes, NJ, USA) using FACSDiva v8.0.2. software (BD, Franklin Lakes, NJ, USA). A total of 300 000 events were recorded to reach the 0.0033% limit of detection and 0.01% limit of quantitation. Analysis was performed with a standard gating strategy, also with FACSDiva v8.0.2. software. Percentage of myeloblasts (CMB-FC%) was calculated as number of cells with myeloblast characteristics (CD117+/CD34+/CD45dim) divided by the number of CD45+ cells. Absolute myeloblast count (CMB-FC#) was calculated in a dual platform scenario; with the following formula: CMB-FC# = (WBC×1000×CMB-FC%) / 100. White blood count (x10^9^/L) was obtained from the automated haematology analyzer Siemens ADVIA 2120i (Siemens Healthineers, Erlangen, Germany). For quality control, the cytometer setup and tracking beads were measured daily to keep the performance tracking of the equipment. An external quality control assessment was also performed by participating in the United Kingdom National External Quality Assessment Service (UK NEQAS) immunophenotyping program.

*Molecular and cytogenetic analysis*

Mutation analysis was performed on DNA from peripheral blood cells. JAK2 V617F, CALR exon 9 and MPL W515 mutations were detected by real-time polymerase chain reaction or high-resolution melting analysis. Next generation sequencing was used to detect high risk mutations (HMR) determined by currently used prognostic scoring systems, previously shown to be prognostically informative in PMF. The panel utilized for next generation sequencing included the following genes: *ABL1, ACD, ANKRD26, ASXL1, BCOR, BRAF, CALR, CBL, CEBPA, CSF3R, CUX1, DDX41, DNMT3A, ERCC6L2, ETNK1, ETV6, EZH2, FLT3, GATA1, GATA2, GNAS, GNB1, IDH1, IDH2, IKZF1, JAK2, KDM6A, KIT, MPL, MYC, NF1, NPM1, NRAS, PDGFRA, PHF6, PRPF8, PTPN11, RAD21, RUNX1, SAMD9, SAMD9L, SETBP1, SETD2, SF3B1, SH2B3, SMC1A, SMC3, SRP72, SRSF2, STAG2, TERC, TERT, TET2, TINF2, TP53, U2AF1, WT1, ZRSR2*. Cytogenetic data were analysed using standard techniques and reported according to the International System for Human Cytogenetic Nomenclature criteria.

| **First author** | **Citation** | **Year** | **MF Related Restriction** | **Therapy Related Restriction** | **No. of Patients** | **Median CMB** | **Min CMB** | **Max CMB** | **Cut-off** | **Associated Inferior Outcome** |
| --- | --- | --- | --- | --- | --- | --- | --- | --- | --- | --- |
| **Visual- or CBC-based analysis** | | | | | | | | | | |
| Barosi G. | [23] | 1988 | - | - | 137 | n/a | 0 | 62 | >24% | OS |
| Visani G. | [24] | 1990 | no post-PV/ET MF | - | 133 | 0 | 0 | 10% | >10% | OS |
| Thiele J. | [25] | 1992 | no post-PV/ET MF | - | 140 | 0 | 0 | 9% | ≥1% | OS |
| Dupriez B. | [26] | 1996 | no post-PV MF | - | 195 | 0-2%* | n/a | n/a | >2% | OS, death from PHT |
| Cervantes F. | [27] | 1997 | no post-PV/ET MF | - | 106 | 0* | 0* | n/a | ≥1% | OS |
| Cervantes F. | [28] | 1998 | age ≤55 years no post-PV/ET MF | - | 121 | <1%* | n/a | n/a | ≥1% | OS |
| Barosi G. | [9] | 2001 | - | - | 73 | 0 | 0 | 19 | >0% | none |
| Okamura T. | [29] | 2001 | - | - | 336 | <3% | n/a | n/a | 3-<5%, ≥5% | OS |
| Sagaster V. | [10] | 2003 | no post-PV/ET MF | - | 110 | n/a | n/a | n/a | n/a | none |
| Arora B. | [11] | 2005 | - | - | 94 | 1% | 0 | 43% | cont. | OS |
| Dingli D. | [30] | 2006 | age <60 years no post-PV/ET MF | - | 58 | ≤2%* | n/a | n/a | >2% | OS |
| Tefferi A. | [31] | 2007 | no post-PV/ET MF | - | 334 | 0 | 0 | 25% | ≥1% | OS |
| Huang J. | [32] | 2008 | no post-PV/ET MF | - | 311 | 0 | 0 | 10.5% | ≥3% | OS, LFS |
| Cervantes F. | [33] | 2009 | no post-PV/ET MF no prefibrotic MF | - | 1018 | n/a | n/a | n/a | ≥1% | OS |
| Tam C.S. | [34] | 2009 | - | - | 370 | <10%* | n/a | n/a | ≥10% | OS |
| Morel P. | [35] | 2010 | no post-PV/ET MF | - | 172 | ≤1%* | n/a | n/a | >1% | OS |
| Tefferi A. | [36] | 2011 | no post-PV/ET MF | - | 884 | 1% | 0 | 18% | 2-<9%, ≥9% | OS |
| Rago A. | [37] | 2015 | no post-PV/ET MF | - | 269 | <1%* | n/a | n/a | ≥1% | LFS |
| Passamonti F. | [8] | 2017 | no primary MF | - | 685 | <3%* | n/a | n/a | ≥3% | OS |
| Guglielmelli P. | [6] | 2018 | age ≤70 years no post-PV/ET MF | - | 805 | <2%* | n/a | n/a | ≥2% | OS, LFS |
| Tefferi A. | [38] | 2018 | age ≤70 years no post-PV/ET MF | - | 311 | n/a | n/a | n/a | ≥2% | OS |
| Tefferi A. | [7] | 2018 | no post-PV/ET MF | - | 641 | <1%* | n/a | n/a | ≥2% | OS |
| Vallapureddy RR. | [39] | 2019 | no post-PV/ET MF | - | 1283 | 0 | 0 | 18 | ≥3% | LFS |
| Mora B. | [40] | 2019 | no primary MF | - | 805 | <3%* | n/a | n/a | ≥3% | LFS |
| **First author** | **Citation** | **Year** | **MF Related Restriction** | **Therapy Related Restriction** | **No. of Patients** | **Median CMB** | **Min CMB** | **Max CMB** | **Cut-off** | **Associated Inferior Outcome** |
| **Visual- or CBC-based analysis (continued)** | | | | | | | | | | |
| Masarova L. | [41] | 2020 | - | - | 1316 | 0* | 0 | 19 | ≥4% | OS, LFS |
| Palandri F. | [42] | 2020 | - | pts. on RUX | 589 | 0 | 0 | 9 | ≥3% | LFS (SMF only) |
| Barosi G. | [13] | 2021 | no post-PV/ET MF | - | 297 | n/a | n/a | n/a | ≥1% | OS |
| Palandri F. | [43] | 2022 | - | pts. on RUX | 794 | 0* | 0 | 9 | 0; 1-4; 5-9% | OS, LFS, RDFS, spleen- & symptom response§ |
| Gangat N. | [44] | 2023 | trial criteriaҰ | pts. on JAKi¥ | 183 | 1 | 0 | 14 | ≥1% | none |
| **Flow-cytometry analysis of circulating CD34+ cells** | | | | | | | | | | |
| Barosi G. | [9] | 2001 | - | - | 84 | 91.6x10^6^/L | 0 | 2460x10^6^/L | >300x10^6^/L | OS, LFS |
| Sagaster V. | [10] | 2003 | no post-PV/ET MF | - | 20 | 36.7x10^6^/L | 0.7 x10^6^/L | 652x10^6^/L | n/a | n/a |
| Arora B. | [11] | 2005 | - | - | 94 | 54.7x10^6^/L | 0 | 5345x10^6^/L | 100x10^6^/L | OS, LFS, PFS† |
| Alchalby H. | [12] | 2012 | - | post allo-Tx | 59 | 94x10^6^/L | 1x10^6^/L | 3461x10^6^/L | 32x10^6^/L | DFS‡ |
| Barosi G. | [13] | 2021 | no post-PV/ET MF | - | 297 | n/a | n/a | n/a | >50×10^6^/L | OS |
| Mannelli F. | [14] | 2022 | - | - | 363 | 21.77x10^6^/L | 0 | 4431x10^6^/L | MFC Score∆ | OS |
| Iurlo A. | [15] | 2023 | - | pts. on RUX | 49 | 83.5x10^6^/L | 1x10^6^/L | 1528x10^6^/L | continous  variable | OS,  spleen length BCM |
| Demoy M. | [16] | 2026 | - | - | 68 | 24.8×10^6^/L | 1.1×10^6^/L | 1892×10^6^/L | ≥100×10^6^/L | OS |

**Supplementary Table 1. Published data regarding the prognostic value of circulating myelobasts in patients with myelofibrosis**

CMB – circulating myeloblast, CBC – complete blood count, MF – myelofibrosis, PV – polycythaemia vera, ET – essential thrombocytosis, SMF – secondary MF, OS – overall survival, LFS – leukemia-free survival, PFS – progression-free survival, DFS – disease-free survival, PHT – portal hypertension, RDFS – ruxolitinib discontinuation-free survival, BCM – below costal margin, Tx – bone marrow transplantation, RUX – ruxolitinib, JAKi – Janus kinase inhibitor, MFC – multiparameter flow cytometry, n/a – not reported

* estimated from published data;

§ according to the International Working Group-Myeloproliferative Neoplasms Research and Treatment (IWG-MRT) criteria

† defined as one of the following occurring after the time of initial peripheral blood (PB) CD34 assessment: a decrease in hemoglobin level by more than 20 g/L or a 100% increase in red blood cell transfusion requirement, emergence of new constitutional symptoms (fever, night sweats and weight loss), a >50% increase in palpable splenomegaly, appearance of PB blasts if previously absent, development of either leukopenia (<4x10^9^/L) or marked leucocytosis (>30x10^9^/L) if previously absent and leukemic transformation (20% or more blast percentage in bone marrow)

‡ not defined

∆ increased CD34+ cell count (>100×10^6^/L) and decreased side scatter (SSC) of neutrophils (<6)

Ұ NCT00935987, NCT00509899, NCT00631462, NCT01420770 and NCT01236352

¥ ruxolitinib, fedratinib, momelotinib and BMS-911543


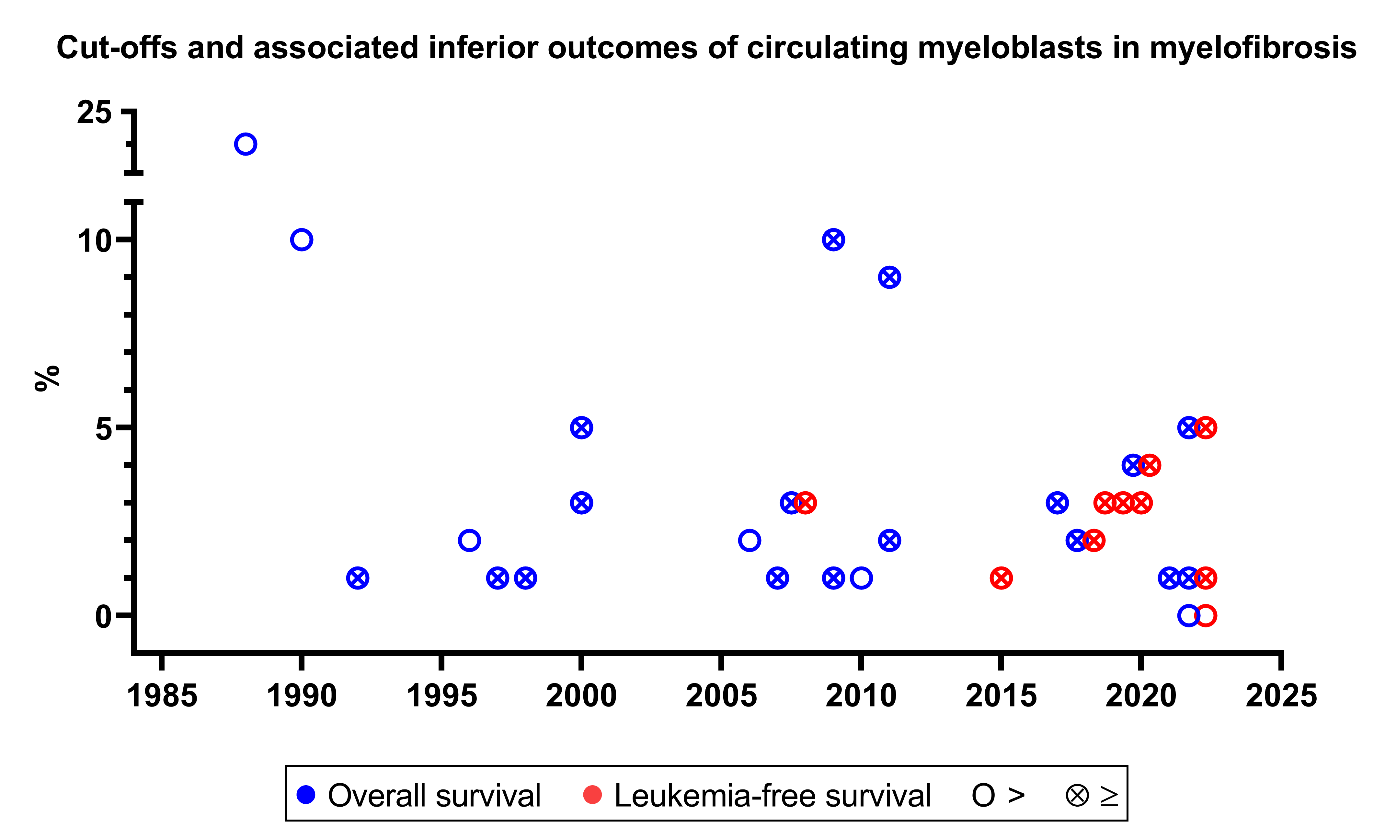


**Supplementary Figure 1. Published data regarding cut-offs and associated inferior outcomes of circulating myeloblasts via visual- or complete blood count-based analysis in myelofibrosis**
